# Runs of homozygosity analysis and genomic inbreeding estimation in Sumba Ongole cattle (*Bos indicus*) using a BovineSNP50K BeadChip

**DOI:** 10.14202/vetworld.2024.1914-1919

**Published:** 2024-08-27

**Authors:** Widya Pintaka Bayu Putra, Hartati Hartati, Redi Aditama, Eko Handiwirawan, Endang Tri Margawati, Simon Elieser

**Affiliations:** 1 Research Center for Applied Zoology, National Research and Innovation Agency (BRIN), Bogor 16911, Indonesia; 2 Research Center for Animal Husbandry, National Research and Innovation Agency (BRIN), Bogor 16911, Indonesia; 3 Department of Agronomy and Horticulture, Faculty of Agriculture, IPB University, Bogor 16911, Indonesia

**Keywords:** BovineSNP50K BeadChip, *Bos taurus* autosomes, F_ROH_, runs of homozygosity, Sumba Ongole

## Abstract

**Background and Aim:**

Runs of homozygosity (ROH) is a biocomputational technique for identifying homozygous regions in the genomics of livestock. This study aimed to determine the ROH in Sumba Ongole (SO) bulls (n = 48) using the BovineSNP50K BeadChip.

**Materials and Methods:**

GenomeStudio 2.0 software was used to generate the BovineSNP50K BeadChip output. The ROH and ROH-based inbreeding coefficients (F_ROH_) were determined using the detect RUNS R v4.1.0 package. Using the following filtering criteria, PLINK v1.90 software was used to perform genotype quality control: (1) Individuals and single-nucleotide polymorphism (SNPs) had call rates >0.95; (2) more than 0.05 was the minor allele frequency; (3) the list contained only SNPs linked to autosomes; and (4) SNPs that strongly deviated (p < 1e-6) from Hardy–Weinberg equilibrium were removed. Subsequently, 25,252 autosomal SNP markers were included in the ROH and F_ROH_ analyses.

**Results:**

In general, the number and length of ROH segments in pool animals were 149.77 ± 16.02 Mb and 486.13 ± 156.11 Mb, respectively. Furthermore, the ROH segments in the animals under study can be discriminated into two classes of 1–4 Mb (83.33%) and 4–8 Mb (16.67%). Subsequently, *Bos taurus* autosomes (BTA) 1, BTA6, and BTA14 had significant homozygous segments comprising 13 genes. Despite this, the average F_ROH_ in pool animals was 0.20 ± 0.06.

**Conclusion:**

These findings indicate that a recent inbreeding event in SO cattle occurred many generations ago. Furthermore, the candidate genes identified from the ROH analysis indicate phenotypic attributes associated with environmental adaptation and economic traits.

## Introduction

Sumba Ongole (SO) cattle, a breed of *Bos indicus* that is indigenous to Indonesia, have demonstrated strong adaptation capabilities on Sumba Island. The introduction of this cattle breed can be traced back to the year 1900, when the breed was brought from India by the Dutch colonial government for use as a draught animal resource [1]. SO bulls can reach an adult weight of 474.08 ± 25.98 kg [2] and a carcass weight of 264.06 ± 14.72 kg at the same adult weight [3]. A genome-wide association study (GWAS) was conducted in SO cattle to determine candidate genes and population structure in these cattle [4, 5]. However, the assessment of GWAS for runs of homozygosity (ROH) in SO cattle has not been reported. ROH represent lengthy uninterrupted segments of homozygous genetic material within the genome, originating from a combination of two identical haplotypes inherited from a common progenitor [6]. Moreover, ROHs serve as crucial resources for investigating genome architecture, particularly in relation to alleles that contribute to genetic enhancement in livestock [7]. The presence of homozygous genomic segments may be influenced by various factors, such as intense selection pressure, historical population dynamics, and consanguinity levels [8]. Recently, ROH analysis has been employed to calculate the ROH-based inbreeding coefficients (F_ROH_) of livestock species [9].

In general, the inbreeding coefficient of livestock is calculated from pedigree data through statistical analysis. However, genome sequences comprising many single-nucleotide polymorphism (SNP) loci have been used to estimate the inbreeding coefficient when the pedigree data are not available [10]. Theoretically, an individual’s inherited allele throughout a genome with hundreds of thousands of loci can be used to directly infer the likelihood of an allele at a specific location [11]. Hence, estimating the inbreeding coefficient using genome information yields accurate results, as reported by Nishio *et al*. [12].

Illumina, Inc. (USA) launched the BovineSNP50K BeadChip, a genome-wide genotyping array for cattle, in partnership with the US Department of Agriculture Agriculture Research Service (ARS), the University of Alberta, and the University of Missouri. With a 49.4–Kb average probe spacing and 54,609 SNP probes, the BeadChip offers more than enough SNP density to support strong genome associations in cattle [13]. Hence, this technique, which includes ROH and F_ROH_ analyses, is important for genomic selection in cattle. Recently, ROH and F_ROH_ analyses have been performed in many breeds of cattle, including Fleckvieh [14], Gyr [8], Wagyu [15], Friesian Holstein [16], Creole [17], Kazakh White-headed, Auliekol [18], and Sahiwal [19]. Since 1900, SO cattle have been kept by farmers on Sumba Island in Indonesia. Therefore, most SO cattle were managed using an extensive management system without recording. Furthermore, none of Indonesia’s numerous artificial insemination centers have generated frozen sperm (straw) from SO bulls. In addition, no scheme is available for sperm distribution. As a result, the inbreeding coefficient of SO cattle can be increased annually, thereby reducing their productivity.

This study aimed to analyze ROH and F_ROH_ levels in SO cattle using the BovineSNP50K BeadChip. The results of this study are important for the genomic selection of SO cattle in the future.

## Materials and Methods

### Ethical approval

The study protocol was approved by the Animal Ethics Committee of the Indonesian Agency for Agricultural Research and Development (Permit number: Balitbangtan/Lolitsapi/Rm/08/2018).

### Study period and location

The study was conducted from June to August 2018 at the SO cattle feedlot farm and the Bubulak slaughterhouse in Bogor, West Java, Indonesia.

### Sample collection, SNP genotyping, and data filtering

The current study’s animal genomics dataset was gathered from 48 SO bulls raised at a feedlot farm (PT. Cahaya Anugrah Gemilang, Bogor, West Java. The animals were genotyped using a BovineSNP50K BeadChip (Illumina, USA) by Macrogen Inc. (South Korea) with the extracted DNA (±50 ng/μL). GenomeStudio 2.0 software (https://support.illumina.com/downloads/genomestudio-2-0.html) was used to generate the BovineSNP50K BeadChip output. Using the following filtering criteria, PLINK v1.90 software (https://www.cog-genomics.org/plink/) [20] was used to perform genotype quality control: (1) Individuals and SNPs had call rates greater than 0.95; (2) more than 0.05 was the minor allele frequency; (3) the list contained only SNPs linked to autosomes; and (4) SNPs that strongly deviated (p < 1e-6) from Hardy-Weinberg equilibrium were removed.

### Detection and classification of homozygosity runs

ROH were computed for each animal using a sliding window in R-4.4.1.software (https://cran.r-project.org/bin/windows/base/). The ROH were calculated using the following parameters [21]: (1) One missing SNP was permitted in the ROH and up to one possible heterozygous genotype; (2) 20 was the minimum number of consecutive SNPs that made up a ROH; (3) there was a minimum density of one SNP every 100 kb; and (4) there was a maximum gap of 1 Mb between consecutive SNPs. This study defined an ROH segment with 100 or more consecutive SNPs as a homozygous segment. Zambrano *et al*. [16] and Liu *et al*. [22] recommended that all identified ROH segments be classified into two distinct classes of 1–4 Mb and 4–8 Mb.

### Common ROH and gene annotation

The number of times each SNP appeared in the ROH was considered and normalized by dividing it by the total number of animals in the analysis to identify genomic areas with high homozygosity. The values were plotted against the SNP location on the chromosome was performed [23]. If not, neighboring SNPs that were more than this criterion eventually combined to form genomic regions of ROH segments, which are shared by most people in the population [24]. The genes in the ROH region were then annotated using the bovine genome of *Bos indicus* (GCF_000247795.1) from the NCBI database (https://ncbi.nlm.nih.gov). Every annotated gene in the ROH region has a biological function deduced from numerous precise literature searches.

Means and standard deviations were calculated using Statistical Package for the Social Sciences 16.0 for Windows (IBM SPSS, NY, USA) packages for the length of ROH segments, total length of ROH segments, genome coverage, number of SNPs per ROH segment, and number of ROH segments.

### F_ROH_

The F_ROH_ was computed in each animal using the formula mentioned by Bjelland *et al*. [25] as follows:


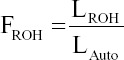


F_ROH_ is the genomic inbreeding coefficient;

L_ROH_ is the entire length of ROH in the genome of an animal.

L_Auto_ is the length of the autosomal genome, *i.e*., 2,671,695,104 bp [26].

## Results

### ROH

The mean length and number of ROH segments per animal are presented in Table-1. The length of ROH segments, total length of ROH segments, genome coverage, and number of SNPs per ROH segments in 4–8 Mb class were higher than in 1–4 Mb class. In contrast, the number of ROH segments in 1–4 Mb class was higher than in 4–8 Mb class.

**Table-1:** Detail of ROH segments per animal in Sumba Ongole cattle.

Parameter	Class		Pool

	1–4 Mb	4–8 Mb	
Length of the ROH segment (Mb)	2.86 ± 0.37	5.41 ± 1.07	3.28 ± 1.10
Total length of the ROH segment (Mb)	429.26 ± 75.20	770.49 ± 145.68	486.13 ± 156.11
Genome coverage (%)	16.07 ± 2.81	28.84 ± 5.45	18.20 ± 5.84
Number of SNPs in each ROH segment	31.25 ± 3.77	45.08 ± 16.99	33.28 ± 8.52
Number of ROH segments	150.98 ± 16.62	143.75 ± 11.57	149.77 ± 16.02

ROH=Runs of homozygosity, SNP=Single-nucleotide polymorphism

**Figure-1:**
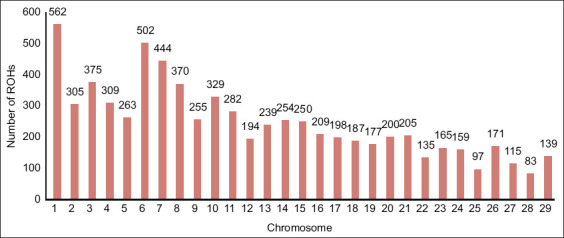
Number of runs of homozygosity segments per chromosome in Sumba Ongole cattle.

**Figure-2:**
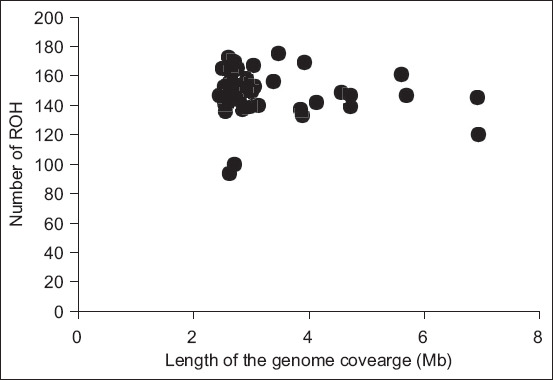
Relationship between the number of runs of homozygosity (ROHs) per individual (dots) and genome length covered by ROHs.

**Figure-3:**
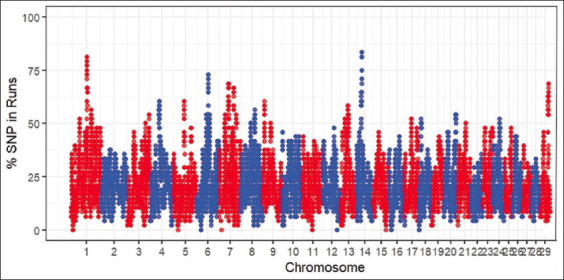
Manhattan plot of the occurrence of single-nucleotide polymorphisms in runs of homozygosity among Sumba Ongole cattle.

Overall, the SO cattle had 3.28 ± 1.10 Mb of ROH segment length, 486.13 ± 156.11 Mb of total ROH segment length, 33.28 ± 8.52 SNPs per ROH segment and 149.77 ± 16.02 ROH segments. In general, the highest number of ROHs per chromosome was observed in *B. taurus* autosomes (BTA)1 (562 segments), followed by BTA6 (502 segments), as shown in Figure-1. Despite this, the total length of ROH segments in the animals under study ranged from 2 to 8 Mb, and the number of ROH segments ranged from 80 to 180, as illustrated in Figure-2. Therefore, a Manhattan plot with a threshold line of 75% revealed many potential SNPs in the BTA1 and BTA14 regions Figure-3.

### Genomic inbreeding and detection of associated genes

The average F_ROH_ of the animals used in this study ranged from 0.10 to 0.40, as shown in Table-2. In general, the F_ROH_ value in the 4–8 Mb class was higher than 1–4 Mb class (0.17 ± 0.03 vs. 0.31 ± 0.06). Therefore, the three loci of BTA had high homozygous SNPs, that is, BTA1, BTA6, and BTA14 (Table-3). However, the total length of ROH segments in BTA6 (687,618 bp) was lower than those in the BTA1 (2,247,563 bp) and BTA14 (1,691,438 bp) regions. Furthermore, a total of 38 candidate genes was detected in the highest frequency of ROH segments (ROH hotspot) and spread to the BTA1 (6 genes), BTA6 (4 genes) and BTA14 (3 genes) regions. Despite this, a total of 56 homozygous SNPs was found at the highest frequency of ROH hotspots and distributed at BTA1 (21 SNPs), BTA6 (12 SNPs), and BTA14 (23 SNPs).

**Table-2:** F_ROH_ of Sumba Ongole cattle.

Class	n	F_ROH_	SD	Minimum	Maximum
1–4 Mb	40	0.17	0.03	0.10	0.27
4–8 Mb	8	0.31	0.06	0.24	0.40
Pool	48	0.20	0.06	0.10	0.40

n=Number of samples, F_ROH_=Genomic inbreeding coefficient, SD=Standard deviation

**Table-3:** Detection of SNPs and genes associated with the highest frequency of ROH segments in the Sumba Ongole cattle genome.

BTA	nSNP	Start	End	Length (bp)	Gene*
1	21	82,176,939	84,424,501	2,247,563	*DGKG*, *ETV5*, *IGF2BP2*, *MAP3K13*, *C1H3orf70*, *VPS8*
6	12	60,391,803	61,079,420	687,618	*KLHL5*, *WDR19*, *RFC1*, *and KLB*
14	23	26,060,343	27,751,780	1,691,438	*RAB2A*, *CHD7*, *CLVS1*,

BTA=*Bos taurus* autosome, nSNP=Number of single-nucleotide polymorphisms per run of homozygosity segment;

* Assembly=*Bos_indicus*_1.0 (GCF_000247795.1)

## Discussion

This study aimed to analyze ROH and F_ROH_ levels in SO cattle using the BovineSNP50K BeadChip. The average length of ROH segments in Pakistani Sahiwal cattle (*B. indicus*) under 4–8 Mb class was approximately 5.66 Mb with 586 ROH segments based on the BovineSNP140K BeadChip [19], which is close to the length in the present study. Despite this, a close length of ROH segment (4–8 Mb class) was reported in Montana beef cattle (*B. taurus*) with approximately 5.62 Mb and 3307 ROH segments based on the BovineSNP30K BeadChip [27]. Furthermore, a similar finding to the present study was shown in Gyr cattle (*B. indicus*) that had 2.77 ± 0.55 Mb (2–4 Mb class) and 5.54 ± 1.12 Mb (4–8 Mb class) of ROH length based on the same BeadChip [8]. In addition, Santos *et al*. [28] obtained the length of an ROH segment (4–8 Mb class) that was close to the length obtained in Curraleiro Pé-Duro (5.75 Mb with 506 ROH segments) and Pantaneiro (5.94 Mb with 62 ROH segments) based on the same BeadChip. In this study, the total length of ROH segment SO cattle was highest compared with many *B. taurus* cattle, such as Friesian Holstein (290.60 ± 67.20 Mb), Polish Red (142.80 ± 67.40 Mb), Limousin (180.50 ± 79.90 Mb), and Simmental (201.80 ± 99.40 Mb) based on the same BeadChip [23]. Subsequently, the number of ROH segments in the studied animals was higher than that in Brown Swiss (94.60 ± 11.60), Marchigiana (71.40 ± 11.10), Piedmontese (54.00 ± 7.20), Friesian Holstein (78.80 ± 9.60), Polish Red (46.40 ± 9.80), Limousin (74.70 ± 9.90), and Simmental (81.50 ± 11.80) based on the same BeadChip [7, 23].

The length of the ROH segment can be classified as small (1–4 Mb), moderate (4–8 Mb), or large (>8 Mb), as described by Liu *et al*. [22]. Very long ROH segments (>20 Mb) are believed to have originated from common autozygosity. In contrast, most short elements are thought to have originated from more distant ancestors, which were the result of intensive selection [21]. Ferencakovic *et al*. [14] explained that the ROH >1, >2, >4, >8, and >16 Mb represent the presence of inbreeding from 50, 25, 12.5, 6, and 3 generations ago in cattle, respectively. In this study, most of the animals had low and moderate lengths of ROH segment (2–5 Mb), which explains the recent inbreeding from about 10–50 generations ago. In this study, the short length of the ROH segments indicated that there were no signals of strong recent selection in the SO cattle. In general, the length and number of ROH segments can be influenced by selection, inbreeding, and the type of BeadChip used for analysis [29].

Compared with other cattle breeds, the F_ROH_ value (>4 Mb) in SO cattle was higher than that in Austrian Fleckvieh (0.03), Brown Swiss (0.10), Friesian Holstein (0.07), Marchigiana (0.05), Piedmontese (0.01), Simmental (0.03), Polish Red (0.03), Limousin (0.03), Hereford (0.10), Charolais (0.04), Montbeliarde (0.08), Kazakh White-headed (0.04), Auliekol (0.02), Caqueteño Creole (0.10), Gyr (0.01), Japanese Black (0.11), and Pakistani Sahiwal (0.02) cattle breeds [7, 8, 12, 14, 17–19, 23]. Despite this, Zambrano *et al*. [16] reported that F_ROH_ value in Friesian Holstein was 0.28, which was close to that of the pool animals under study. Furthermore, the F_ROH_ values in native Chinese cattle breeds were close to those of SO cattle (1–4 Mb class) *i.e*. 0.1 for Leiqiong; 0.12 for Lufeng; and 0.15 for Hainan [30].

In Modicana cattle (*B. taurus*), many candidate genes were detected in BTA1 (25 genes/39 homozygous SNPs) and BTA6 (25 genes/112 homozygous SNPs) according to ROH analysis [31]. Despite this, Santos *et al*. [28] reported that BTA6 and BTA14 in two Italian cattle breeds (Curraleiro Pe-Duro and Pantaneiro) had high-length ROH segments, but they did not identify candidate genes in either region. Subsequently, Zambrano *et al*. [16] identified candidate genes for BTA1 (six genes) and BTA6 (five genes) in the Friesian Holstein cattle of Colombia based on ROH analysis.

Previous studies by Viale *et al*. [32], Persichilli *et al*. [33], Passamonti *et al*. [34] and Hiltpold *et al*. [35] reported that many candidate genes detected in Table-3 were associated with body conformation (*RAB2A*), milk production (*DGKG, VPS8*), and reproduction (*MAP3K13, WDR19*) in cattle. Hence, the candidate genes selected from the ROH analysis in the present study were the main functional genes involved in economic traits and environmental adaptation in the tropical climate of Indonesia. Moreover, genetic improvements to adaptability and survivability traits are important when climate changes can influence the economic traits of cattle [36].

## Conclusion

The findings of this study indicate that a recent inbreeding event in SO cattle occurred many generations ago. Furthermore, the candidate genes identified through ROH analysis indicate phenotypic attributes associated with environmental adaptation and economic traits.

Several factors may influenced this research, such as, sample size, BovineSNP50K BeadChip resolution, environmental and management factors, historical data that affect the genetic variants of the population and limit the interpretation of the results of ROH analysis and inbreeding estimates.

Assessing the whole-genome sequencing (WGS) or SNP chips with more markers, comparative studies with other *Bos indicus* populations, long-term studies combining genomic data with environmental and livestock management information to understand inbreeding dynamics and its impact on productivity, conservation strategies to preserve genetic diversity and reducing the risk of inbreeding are some of the scopes of research that can be carried out in the future.

## Authors’ Contributions

HH: Designed the study, collected data, performed fieldwork, supervised the study, and prepared and revised the manuscript. WPBP, RA, EH, ETM, and SE: Designed the study, analyzed data, and prepared and revised the manuscript. All authors have read, reviewed, and approved the final manuscript.
